# On the Structure and Uses of the Spleen

**Published:** 1808-09

**Authors:** Everard Home


### ON THE STRUCTURE AND USES OF THE SPLEEN.

By Everard Home, Esq. F. R. S.

[ From the last Number of the Philosophical Transactions. ]

In bringing forward a fact of so much importance, as a communication between the cardiac portion of the stomach and the circulation of the blood, through the medium of the spleen, I shall not take up the time of the Society by offering any preliminary observations, but state the circumstances which led to the discovery, and the experiments by which the different facts have been ascertained.

During the investigation of the functions of the stomach, (in which I have been lately engaged,) it was found that while digestion is going on, there is a separation between the cardiac and pyloric portions, either by means of a permanent or muscular contraction. This fact placed the process of digestion in a new light, and led me to consider in what way the quantities of different liquors, which are so often taken into the stomach, can be prevented from being mixed with the half digested food, and interfering with the formation of chyle.

Pursuing this inquiry, I found that the fluids are principally contained in the cardiac portion, and the food that has reached the pyloric portion is usually of one uniform consistence, so that the fluids beyond what are necessary for digestion, would appear to be carried out of the stomach, without ever reaching so far as the pylorus. To ascertain the truth of this opinion is the object of the present paper.

The lymphatic vessels of the stomach are numerous, but they are equally or more so in the other viscera. Many circumstances appeared to render it probable, that the spleen is the route by which liquids are conveyed. The more I considered the subject, new reasons in favour of this opinion crowded on my mind, so as almost to enforce conviction, and made me set about devising various methods, by which its truth or falshood might be established.

The first point to be decided was, whether the liquids received into the stomach do escape in any considerable quantity, when prevented from passing out at the pylorus. This was ascertained by the following experiment, made October 31, 1807, with the assistance of Mr. Brodie, Mr. W. Brande, and Mr. Clift.

The pylorus of a small dog was secured by a ligature, and a few minutes afterwards five ounces by measure, of an infusion of indigo in water, of the temperature of the atmosphere, were injected by the mouth into the stomach. At the end of half an hour the dog became sick, and brought up by vomiting two ounces of a nearly colourless fluid. The dog was immediately killed, and the different parts were examined. The pylorus was found completely secured by the ligature, so that nothing could pass in that direction. The pyloric portion of the stomach was found empty and contracted; the cardiac portion contained about two ounces of solid contents, enveloped in a gelatinous substance, and one ounce of water with little or no colour, the indigo being completely separated from it, and spread over the surface of the internal membrane. Of the five ounces of water thrown into the stomach, two were brought up by vomiting, and one only remained; two ounces had therefore escaped in the course of half an hour. As the stomach contained two ounces of solid food at the time the experiment was made, it is reasonable to suppose that there was also some liquid in it, and in this case the whole quantity that escaped must have exceeded two ounces. On examining the external covering of the stomach, and along the course of the vasa brevia, where the absorbents ususally pass, none were discovered, so that these vessels were not at that time carrying any liquid.

The spleen was turgid, unusually large, and its external surface very irregular; when cut into, small cells were every where met with, containing a watery fluid, and occupying a considerable portion of its substance. This appearance, which I had never seen before, made me enquire, if it had been taken notice of by others, and endeavour to ascertain the circumstances under which it is produced. The following statement contains the information which I have received on this subject.

Malpighi appears to be the first anatomist, who had any particular knowledge of the structure of the spleen. He describes its capsule, and a net-work which pervades every part of the substance. He mentions a number of small glands, which are hollow, and surrounded by arterial zones, but he had never been able to trace any venal branches into them. He believed that there was a cellular structure in the spleen containing red blood, interposed between the arteries and veins; this led him to adopt a theory that the net-work was muscular, and by its action propelled propelled the blood, so that there was a systole and diastole in the spleen, as in the heart.

STUKELY, in his Gulstonian lecture, has very closely copied MALPIGHI, without giving any additional information.

CUVIER, the latest writer on the subject, in his *Leçons d' Anatomie comparée*, corrects the error of MALPIGHI respecting the nature of the net-work, which he states to be composed of elastic ligament, and says that there are small corpuscles, whose use is unknown, and which disappear when the blood vessels are minutely injected.

In the course of the present investigation, I have examined the spleen after death, under the ordinary circumstances, and have found the appearances described by CUVIER. I have also examined it frequently immediately after the stomach had received unusual quantities of liquids, and in that state have found invariably, that the corpuscles of CUVIER, which were the glands of MALPIGHI, are distinct cells, containing a fluid, which escapes when the cells are punctured, and renders their membranous coat visible, so that it would appear that the distension of these cells is connected with the state of the stomach, and therefore only takes place occasionally; and that the elastic capsule by which the spleen is surrounded adapts the organ to these changes in its volume.

On examining further into the structure of the spleen, in which I have been materially assisted by Mr. BRODIE, the following facts have been ascertained.

In the spleen of the bullock, horse, and hog, the cells, when the arteries and veins are injected with coloured size, are seen to have numerous arterial branches ramifying in their coats, but no venal ones, which confirms the statement of MALPIGHI; and when the cells are empty and contracted, and the blood-vessels filled to a great degree of minuteness, the appearance of cells is entirely lost, as stated by CUVIER.

When the cells were in a distended state, their cavities in a great many instances were very distinct, having been laid open in making a section of the spleen. The intermediate parts of the spleen are but sparingly supplied with arterial branches, and the smaller ones do not appear to have any particular distribution.

When the veins only are injected their branches appear more numerous, and larger than those of the arteries, making the whole substance of the spleen of a red colour. They appear to arise from the outside of the cells, going

 off off at right angles to their circumference, like radii. Where the injection has not been very minute, they are seen to arise at so many points of the capsule; but where the injection has got into smaller branches, their number is so much increased that they appear to form plexuses round the cells.

The trunk of the splenic vein, compared with that of the artery, when both are filled with wax, is found to be in the proportion of five to one in its size. This was ascertained both by an accurate measurement of their diameters, and by weighing half an inch in length of each in a very nice balance; the disproportion between them is greater, than between corresponding veins and arteries in other parts of the body.

Having acquired this knowledge of the internal structure of the spleen, I made the following experiment with a decoction of madder. The substance was employed, from the animals who feed on it having their bones tinged with red, so that there can be no doubt of its colouring matter being carried into the circulation of the blood. I was much disappointed on seeing the colour of the decoction, which, instead of being a bright red, (the tinge communicated to the bones) was of a dirty brown. The same gentlemen assisted me, as in the former experiment.

Nov. 8, 1807, seven ounces of a strong decoction of madder were injected into the stomach of a dog, immediately after the pylorus had been secured. At this time the dog voided some urine, which was limpid and colourless. In 42 minutes, 2 ounces of a yellowish fluid were brought up by vomiting. In 18 minutes more the dog vomited again; what came up proved to consist of 3½ ounces of solid matter, and 3 ounces of liquid. In 15 minutes afterwards, 5 ounces of the decoction were injected, which remained quietly on the stomach for two hours and a quarter, at the end of which period the dog was killed. In the act of dying he made water, in the quantity of two ounces, of a dark muddy colour. This was saved, and afterwards compared with the remaining liquid in the stomach, which it exactly resembled. On examining the connections between the stomach and the spleen, none of the absorbent vessels were apparent, more than in the former experiment. The pyloric portion of the stomach contained about two ounces of half digested food, but no liquid. The cardiac portion contained four ounces of liquid, and half an ounce of solid food, so that the act of vomiting, which appeared, at the time, a sufficient exertion tion to have completely emptied the stomach, had brought up no part of the contents of the pyloric portion, and had not even completely emptied the cardiac portion. In this experiment, without making allowance for any liquid in the stomach, prior to the decoction of madder being injected, one-fourth part of the quantity thrown in had escaped. The cells of the spleen were more distinctly seen than in the former experiment, particularly at the great end.

Although there was every reason to believe that the colouring matter of the madder had been conveyed into the urinary bladder, yet so muddy and indistinct was the colour, that it was by no means completely ascertained. I therefore resolved in my future experiments, to make use of some colouring substance, the presence of which could be detected in a very diluted state, by means of a chemical test; and I requested Mr. W. Brande, of whose assistance I have before availed myself, to point out the substances best fitted for this purpose. He immediately suggested that rhubarb was a substance which he had made use of as a test to ascertain the presence of alkali, and therefore had no doubt that the caustic alkali would prove a test of rhubarb. This substance has also another advantage; it is well known to pass very readily by the kidneys, without being decomposed.

The following are the results of experiments made with rhubarb, to ascertain the best modes of detecting it in the urine and blood, and the time it takes to pass from the stomach to the urinary bladder.

Five drops of tincture of rhubarb added to 3 ounces of water, are found to strike an orange tint when the test is added, which does not take place when the rhubarb is more diluted.

Six drops of tincture of rhubarb, added to three ounces of serum, are readily detected by the eye, but the colour is not heightened by applying the test; the alkali contained in the serum being sufficient to strike as bright a tint, as that quantity of rhubarb can receive from the addition of alkali.

When tincture of rhubarb is mixed with blood just taken from the arm, its colouring matter is afterwards found both in the serum and in the coagulum.

When blood is drawn from the arm of a person, who has taken rhubarb in sufficient quantity to affect the urine, the serum is found to have a slight tinge from it, equal to

 that that, which one drop of tincture of rhubarb gives to half an ounce of serum when added to it.

Half an ounce of tincture of rhubarb, diluted in 1 ½ ounce of water, taken in the interval between meals, did not pass off by urine in less than an hour, and even then was not in sufficient quantity to be discovered till the test was applied.

The same quantity was taken immediately before a breakfast consisting of tea. In 17 minutes, half an ounce of urine was voided, which when tested had a light tinge. In 30 minutes another half ounce was made, in which the tinge was stronger; and in 41 minutes a third half ounce was made, in which it was very deep. In an hour and ten minutes 7 ounces were voided, in which the tinge of rhubarb was very weak, and in two hours twelve ounces were voided, in which it was hardly perceptible.

In 6 ½ hours the rhubarb acted on the bowels, and gave a decided tinge to the fæces; the urine made at the same time had a much stronger tinge, than what was voided at one hour and ten minutes.

In this experiment, the rhubarb appeared to have escaped from the cardiac portion of the stomach; and in two hours ceased to pass through that channel; but was afterwards carried into the system from the intestines, and again appeared in the urine.

This experiment was repeated on another person; the rhubarb was detected in the urine in 20 minutes. In 2 hours the tinge became very faint; in 5 hours it was scarcely perceptible; in seven hours the rhubarb acted on the bowels; and the urine made after that period, became again as highly tinged as at first.

It was suggested by a chemical friend, that the prussiate of potash might be a better substance than rhubarb, for the present experiments, since the solution of one quarter of a grain in two ounces of water, becomes of a blue colour on the addition of the acidulous muriate of iron.

To determine this point, one quarter of a grain was dissolved in two ounces of serum, but no blue colour was produced by the addition of the test, nor did this effect take place till the quantity of the prussiate was increased to a grain; so that minute quantities of the prussiate of potash, or at least of the prussic acid, may exist in the blood, without being detected by adding solution of iron.

The effects of rhubarb on the urine, and the different parts of the blood having been thus ascertained, a third experiment was made, in which that substance was employed, ployed, and I had the assistance of the same gentlemen as in the others.

On November 17, 1807, at 35 minutes past 11 o'clock, five drachms of a mixture of tincture of rhubarb and water, in the proportion of a drachm to an ounce, were injected into the stomach of a dog, whose pylorus was secured. At 20 minutes past 1, two ounces of fluid were brought up by vomiting: ten minutes afterwards, another ounce of the mixture was injected, as were nine drachms more at ½ past 4 o'clock. The two last portions were retained, and at 8 o'clock in the evening the dog was killed.

On examining the parts after death, the pylorus was found to be completely secured; the stomach contained about two ounces of fluid; none of the absorbent vessels passing from its great curvature were in a distended state so as to be rendered visible. The spleen was turgid as in the former experiment, and the urinary bladder full of urine.

This urine tested by the alkali, received a deeper tinge of rhubarb than the human urine, after rhubarb had been taken three hours by the mouth, and in other respects resembled it.

When the spleen was cut into, the cells were particularly large and distinct. A portion of it was then macerated in two drachms of water for ten minutes in a glass vial. All the parts were exposed to the water, by its being divided in all directions. The water thus impregnated was strained off and tested by the alkali, and immediately the reddish brown colour was produced in the centre, and no where else, but in less than a minute it began to diffuse itself, and extended over the whole.

A similar portion of the liver was treated in the same way, and the alkali was added to the strained liquor, but no change took place in it whatever.

In this experiment the rhubarb was detected in the juices of the spleen as well as in the urine; and as there was no appearance of it in the liver, it could not have arrived there through the medium of the common absorbents carrying it into the thoracic duct, and afterwards into the circulation of the blood.

The results of the experiments already brought forward having established the fact that fluids received into the stomach, when the pylorus is closed, pass through the spleen into the circulation of the blood; it became an object to

 determine, determine, by experiment, whether this takes place when the parts are in a natural state.

The ass appeared, on many accounts, the best subject for this purpose; and as it is made use of to teach the veterinary pupils the anatomy of that tribe of animals, I applied to the Professor for permission to make my experiments in the theatre of the college.

This was granted me in the most obliging manner; the subjects were also supplied by the College; and Mr. SEWELL, the assistant Professor, gave me his personal aid with a degree of zeal and ability I have rarely met with, and have much pleasure in acknowledging.

In making the following experiments, I had the assistance of Mr. SEWELL, Mr. BRODIE, Mr. WILLIAM BRANDE, and Mr. CLIFT.

*Experiment* 1. An ass, which had been kept twenty-four hours without hay, to prevent the liquor that was to be poured into its stomach from being soaked up and retained there, on the evening of the 3d of December, 1807, had a drench given it, consisting of half a pint of the spirituous tincture of rhubarb, diluted in half a pint of water. On the morning of the 4th, this was repeated at eight o'clock, and again at twelve. At two o'clock the animal was pithed, so as to destroy its sensibility, and before the circulation was entirely stopped, six ounces of blood were taken from the splenic vein into a graduated glass measure, and a similar quantity was taken from the left auricle of the heart, into a vessel of the same kind: these were allowed to coagulate and separate their serum.

The spleen was large and turgid; upon making sections of it, the cells were found to be very numerous; and towards the great end and near the edge, they were particularly distinct to the naked eye. The cut surface had a strong smell of rhubarb, and when it was applied to white paper wetted with the alkaline test, an orange tinge was produced. This was strongly contrasted by a stain made in the same manner with a section of the liver, which had no such tinge, nor did the liver give the slightest smell of rhubarb.

Infusions were made of the spleen and liver under similar circumstances; these were strained off into separate glasses, and tested by the alkali. The urine was tested in the same way. The serum, from the different portions of blood, was also poured off into separate glass vessels, to which the test was added. In nineteen hours after the blood had been taken from the viens, they were all compared pared together. The urine had so deep a tinge, that it nearly resembled the pure tincture of rhubarb in appearance; the others had a tinge, although in very different degrees; the quantity of rhubarb they contained was estimated by adding tincture of rhubarb to alkaline water so as to produce corresponding tints. The infusion of spleen had a tint equal to sixty drops of tincture of rhubarb in two ounces of alkaline water.: the serum of the splenic vein to fifteen drops: the serum from the left auricle of the heart, to three drops. The infusion of the liver gave no orange tinge, but had it not been obscured by the red particles of the blood, it must have been equal to that of the serum from the auricle.

The connecting membrane between the stomach and spleen was attentively examined, very few absorbent vessels were seen, and these were not in a turgid state; they were traced to the chain of glands situated near the edge of the spleen, which receive the absorbents of the stomach, but none were detected passing beyond the glands, nor did the glands admit quicksilver to pass through them towards the spleen.

*Exp.* 2. The former experiment was repeated upon another ass, with similar results, but less strongly marked; the cause of this difference was explained by the abdominal viscera being in an inflamed state.

The urine was less impregnated with rhubarb, the infusion of the spleen had a lighter tinge, and the serum of the splenic vein had it in a still less degree; but evidently exceeding that of the serum from the vena cava inferior, opened just below the diaphragm, which was substituted for the left auricle of the heart, with a view to vary the experiment.

*Exp.* 3. The same experiment was made on a third ass with similar results.

*Exp.* 4. An ass that had been kept four days without water, and two without solid food, on the evening of the 8th of January, 1808, had a ball given it, containing half an ounce of powdered rhubarb; on the 9th, at seven o'clock in the morning, this was repeated; a third was given at nine o'clock, and a fourth at twelve. At two o'clock the ass was pithed, and four ounces of blood were taken from the splenic vein, and the same quantity from the left auricle of the heart.

The spleen was found contracted to half the size of those in the former experiments; when cut into, the cells were small, and it required a magnifying glass to see them distinctly tinctly. The substance was compact, and bore a near resemblance to a portion of liver; so that in this state the blood vessels, particularly the veins, must have been much contracted in their diameters.

The stomach contained about two ounces and a half of a gelatinous substance mixed with rhubarb, the small intestines were nearly empty, but the cæcum and colon contained several quarts of water, in which the rhubarb was more evident both to the sight and smell, than in the stomach.

The absorbent glands upon the edge of the colon were ranged in two rows, one on each side of the great vein, and were exceedingly numerous. In the space between these rows of glands, in some places twenty trunks of absorbent vessels could be readily counted, of a very large size.

The urine was impregnated with rhubarb, so as to acquire an orange tinge from the addition of the test; but the infusion of the spleen, and the serum of the different portions of blood, did not contain it in sufficient quantity to have the colour heightened by alkali.

*Exp.* 5. The last experiment was repeated upon another ass. Two ounces of blood were taken from the splenic vein, two from the large vein of the colon, and two from the inferior vena cava in the lower part of the loins.

The spleen had the same appearance as in the last experiment.

The stomach contained nearly a pint of moderately solid contents, in which the rhubarb was very evident. The small intestines were nearly empty; but the cæcum and beginning of the colon contained several quarts of liquid, strongly impregnated with rhubarb.

The absorbent glands and vessels had the same appearance as in the former experiment.

The urine when tested was found impregnated with rhubarb.

The portions of serum of the blood taken from these different veins, when tested by the alkali, appeared to be very much alike; at least that from the splenic vein was not more tinged than the others.

*Exp.* 6. Having been informed by Mr. Sewell, that spirituous liquors given in large quantities to horses, produce inflammation of the brain, and sometimes death, and this information having been in some measure confirmed by an ass in a weakly state, that had taken half a pint of the spirituous tincture of rhubarb in the evening, dying in the night, I thought it right to make a comparative rative experiment with the infusion of rhubarb, to determine whether the result would be the same as with the tincture.

February 9, 1808. An ass had a pint of infusion of rhubarb given to it in the evening; the same dose was repeated at six o'clock in the morning of the 10th; and again at nine o'clock, and at twelve. At two o'clock the animal was pithed, and two ounces of blood were taken from the splenic vein, two from the vein of the colon, and two from the inferior vena cava in the lower part of the loins.

The spleen was found turgid, and large; when the cut surface was rubbed on white paper, the orange tint was very evident without any test applied to it, particularly so, when compared with a similar stain made by a section of the liver, in which there was no such tinge.

In the stomach and duodenum, the rhubarb was found in large quantities; but none was met with in the cæcum.

The urine was impregnated with rhubarb, the orange tint upon the application of the alkali being very distinct.

At the end of twenty hours, the serum of the splenic vein had a tinge equal to four drops of the tincture of rhubarb in two ounces of alkaline water; that of the vein of the colon and vena cava was less distinct.

The effects of the infusion of rhubarb on the spleen, the serum of the blood and the urine, corresponded exactly with that of the tincture in the former experiments, but was in a less degree of intensity.

In the course of these experiments, an attempt was made to ascertain whether the blood in the splenic vein has a greater proportion of serum than in the other veins of the body, and the general results were in favour of such an opinion; but it will appear from what follows, that the quantity of serum separated in twenty-four hours, is by no means a just criterion of the proportion which the blood contains.

*Experiment* 1. Three ounces of blood from the arm of a healthy person were received into a graduated glass vessel, previously cooled to the temperature of 32°, three more into a second glass of the temperature of 50°, and three into a third at 70°. The three glasses were brought into a room, the temperature of which varied from 40° to 50°. At the end of nineteen hours, the serum was found in the following quantities.

| | | |
|---|---|---|
| In the glass at | 32° | 9 drachms. |
| | 50° | 11 |
| | 70° | 10 |

The

The blood did not flow so freely into the glass at the highest temperature, as into the other two.

*Exp.* 2. This experiment was repeated, and the serum examined at the end of forty-three hours.

| | | |
|---|---|---|
| In the glass at | 32° | 12 drachms. |
| | 50° | 12 |
| | 70° | 13 |

*Exp.* 3. It was repeated, and the serum examined at the end of 67 hours.

| | | |
|---|---|---|
| In the glass at | 32° | 11 drachms. |
| | 50° | 11 ½ |
| | 70° | 11 ½ |

*Exp.* 4. It was repeated, and the serum measured at the end of ninety hours.

| | | |
|---|---|---|
| In the glass at | 32° | 11 ½ drachms. |
| | 50° | 13 |
| | 70° | 10 ½ |

The blood did not flow so readily into the glass at the highest temperature as into the other two.

From the experiments, it appears that the serum separates in larger quantity, when the blood is received into a vessel at the temperature of 70°, than at 50° or 32°; this, however, is prevented from taking place by the blood not flowing readily from the vein.

From these experiments on the spleen contained in this, and the foregoing part, the following facts appear to have been ascertained.

That the spleen is met with in two very different states, one which may be termed the distended, the other the contracted, and that in the one its size is double what it is in the other. In the distended state there is a distinct appearance of cells containing a limpid fluid, distinguishable by the naked eye; in the contracted, these only become distinct when seen through a magnifying glass. The distended state takes place when the stomach has received unusual quantities of liquids before the animal's death; and the contracted state, when the animal has been kept several days without any drink before the spleen is examined.

That the trunk of the splenic vein (of the hog) is more than five times the size of the trunk of the splenic artery.

That when the pylorus is secured, coloured liquids pass from the cardiac portion of the stomach into the circulation of the blood, and go off by the urine; and while this is going on, the spleen is in its most distended state, and the colouring matter is found in its juices, although it is not not to be detected in those of the liver. The colouring matter cannot therefore be conveyed to the spleen through the common absorbents of the stomach, which lead to the thoracic duct.

That when the pylorus is open, the colouring matter under the circumstances above mentioned is equally detected in the spleen.

That when the spleen is in this state, the blood in the splenic vein has its serum more strongly impregnated with the colouring matter, than that of the blood in the other veins of the body; and when the stomach is kept without liquids, although colouring matter is carried into the system from the intestinal canal by the ordinary channels, no particular evidence of it is met with in the spleen or its veins.

That the cæcum and the portion of the colon immediately beyond it, is found (in the ass) to be at all times filled with liquids, even when none has been received into the stomach for several days, and there is a greater number of absorbent vessels for carrying liquids from the colon into the thoracic duct, than from any other part of the body. The colon is therefore a reservoir, from which the blood vessels are occasionally supplied with liquids.

Mr. Sewell informs me, that the same observation applies in a still greater degree to the horse.

That coloured liquids taken into the human stomach, under some circumstances, begin to pass off by urine in seventeen minutes, continue to do so for some hours, and then disappear; they are again met with in the urine, after the colouring matter is known to have arrived at the great intestines, by its passing off by the bowels.

From the above facts, the following conclusions may be drawn.

That the liquids received into the stomach beyond what are employed for digestion, are not wholly carried out of it by the common absorbents of the stomach, or the canal of the intestines, but are partly conveyed through the medium of the spleen into the circulation of the liver.

The vessels which communicate between the stomach and the spleen have not been discovered; but if it is proved that the colouring matter of the contents of the stomach, is met with in greater quantity in the spleen and in the vein which goes from that organ to the liver, than in the other veins of the body, there appears to be no other mode in which it can arrive there, but by means of such vessels; and the two different states of the spleen, which which correspond with the quantities of liquids that pass from the stomach, are strongly in favour of the existence of such a channel.

This communication between the cardiac portion of the stomach, and the spleen, will explain the circumstance of those who are in the habit of drinking spirituous liquors having the spleen and liver so frequently diseased, and the diseases of both organs being of the same kind.

This organ is not essential to life, its office being of a secondary kind; but when it is materially diseased, or entirely removed, digestion must be disturbed. The extent to which this takes place, cannot be accurately known from experiments on quadrupeds, and the instances in which the human spleen has been removed have not been attended to with sufficient accuracy to afford an explanation of the effects that were produced on the stomach.

